# Characterising deviation from treat-to-target strategies for early rheumatoid arthritis: the first three years

**DOI:** 10.1186/s13075-015-0562-0

**Published:** 2015-03-08

**Authors:** Nasir Wabe, Michael J Sorich, Mihir D Wechalekar, Leslie G Cleland, Leah McWilliams, Anita Lee, Llewellyn Spargo, Robert G Metcalf, Cindy Hall, Susanna M Proudman, Michael D Wiese

**Affiliations:** School of Pharmacy and Medical Sciences and Sansom Institute for Health Research, University of South Australia, GPO Box 2471, Adelaide, 5001 Australia; School of Medicine, Flinders University, GPO Box 2100, Adelaide, SA 5001 Australia; Department of Rheumatology, Royal Adelaide Hospital, North Terrace, Adelaide, SA 5000 Australia; Discipline of Medicine, University of Adelaide, Adelaide, SA 5005 Australia

## Abstract

**Introduction:**

Treat-to-target (T2T) strategies using a protocol of pre-defined adjustments of disease-modifying anti-rheumatic drugs (DMARDs) according to disease activity improve outcomes for patients with rheumatoid arthritis (RA). However, successful implementation may be limited by deviations from the protocol. The aim of this study was to determine the prevalence of protocol deviation, explore the reasons and identify subsets of patients in whom treatment protocols are more difficult to follow.

**Methods:**

In this retrospective cohort study, treatment-naïve patients with RA of less than one year’s duration, attending a dedicated early arthritis clinic between 2001 and 2013, were followed for three years from initiation of combination therapy with conventional DMARDs which was subsequently modified according to a T2T protocol. At each clinic visit, whether deviation from the protocol occurred, the type of deviation and the reasons for deviation were assessed. The relationship between protocol deviations and baseline variables was determined using linear regression analysis.

**Results:**

In total, 198 patients contributed 3,654 clinic visits. The prevalence of protocol deviations was 24.5% and deviation in at least at one clinic visit was experienced by 90.4% of patients. The median time to first deviation was 30 weeks. Continuing existing treatment rather than intensifying therapy was the most common type of deviation (59.9%). Patient and physician related factors were the most common reasons for deviation, each accounting for 24.7% of deviations, followed by toxicities (23.3%) and comorbidities (20.0%). The prevalence of protocol deviations was lower among patients who achieved remission after three years (13.1%; 162 deviations out of 1,228 visits) compared with those who were not in remission (30.9%; 523/1692) (*P* <0.0001). On multivariate analysis, only body mass index (*P* = 0.003) and helplessness score (*P* = 0.04) were independent predictors of protocol deviations although the predictive power of the model was not strong (*R*^*2*^ = 0.17).

**Conclusions:**

Deviation from a T2T protocol occurred in one quarter of visits, indicating that applying the T2T approach is feasible in clinical practice. Failure to escalate dose when indicated was commonly encountered, and just under half of the observed deviations were related to either toxicities or comorbidities and were therefore justifiable on clinical grounds.

## Introduction

Treating rheumatoid arthritis (RA) with disease modifying anti-rheumatic drugs (DMARDs) according to a treat-to-target (T2T) strategy is more effective than traditional routine care [1-5]. In order to achieve rapid disease control, disease activity is assessed regularly and drug doses are escalated or new medications are added until a predefined target disease activity state such as remission or low disease-activity (LDA) is reached [6], in a manner similar to management of other chronic diseases such as diabetes and hypertension [7].

To date, little is known about deviation from T2T recommendations and the reasons for these deviations [8,9]. As the T2T strategy involves frequent assessments of disease activity [10] and application of predefined dose modification criteria (DMC), it may not be feasible outside the clinical trial setting, or the benefits reported in trials may not be realised in the clinic. Adherence to T2T recommendations could be influenced by factors relating to the patient, the physician, the disease, medication or other co-morbid conditions. For example, a physician could deviate from T2T recommendations if his or her assessment of a patient’s disease activity status differed from that determined by composite disease activity measures [9] or patient preference could influence willingness to change therapy when indicated [9-11]. Irreversible joint damage, contraindications and/or past adverse drug reactions to DMARDs, comorbidities and logistic issues are recognised barriers to changing treatment in clinical practice [8].

Contemporary T2T protocols vary from setting to setting. One or more classes of drugs, conventional DMARDs, corticosteroids and biological agents could be included, and the initial approach could be step-up, step-down or parallel therapy. Assessing the extent of deviation from a T2T protocol and the related factors would facilitate an evaluation of the feasibility of the proposed recommendations in clinical practice and increase awareness of common reasons for protocol deviations.

The aims of this study were to determine the prevalence of deviations from a T2T protocol in an early arthritis clinic in which treatment-naïve patients with RA of less than 12 months duration were treated with combination therapy with conventional DMARDs. The type of deviations and the reasons for them were examined and baseline factors associated with deviation were identified.

## Methods and patients

### Setting and study design

Between September 2001 and December 2013, consecutive patients attending the Royal Adelaide Hospital Early Arthritis Clinic were included if they were diagnosed with RA according to the 1987 revised American College of Rheumatology (ACR) [12] or 2010 ACR/European League Against Rheumatism (EULAR) criteria [13], and were 18 years of age or older, with active disease (less than one year duration) and DMARD-naïve. Those with less than one year of follow up were excluded from this analysis, and data for each patient over three years of follow up were analysed. Ethics approval was obtained from the Royal Adelaide Hospital Research Ethics Committee and patients gave informed consent.

### Initial treatment and follow up

All patients initially received triple therapy with methotrexate (MTX) (10 mg/week), hydroxychloroquine (HCQ) (400 mg daily) and sulfasalazine (SSZ) (500 mg/day increasing to 2 g over 4 weeks) (Table 1). Exceptions were patients who were sensitive to sulphur-containing compounds (where sulfasalazine was omitted) and/or any other disease known to increase the toxicity of these DMARDs. Disease activity status was assessed at clinic visits every 3 to 6 weeks in the first three months then every 6 weeks until the criteria to escalate dose (see below) were no longer fulfilled. Modification of therapy for toxicity occurred at any time as clinically indicated. Parenteral administration of corticosteroids was permitted to temporarily reduce disease activity if required, and the use of oral corticosteroids and non-steroidal anti-inflammatory drugs was actively discouraged.

**Table 1:** **Algorithm for treat-to-target disease-modifying anti-rheumatic drug** (**DMARD) therapy**

Follow up, weeks*	**DMC fulfilled** ^**1**^	**Medications**			
		**Methotrexate (MTX)**	**Hydroxychloroquine (HCQ)**	**Sulfasalazine (SSZ)**	**Other DMARDs**
**0**		10 mg/wk oral^2^	400 mg/d	0.5 g/d to 2.0 g/d^3^	
**6**	Yes	10 mg/wk oral^2^	400 mg/d	3.0 g/d	
**12**	Yes	15 mg/wk oral^2^	400 mg/d	3.0 g/d	
**18**	Yes	20 mg/wk oral^2^	400 mg/d	3.0 g/d	
**24**	Yes	25 mg/wk oral^4^	400 mg/d	3.0 g/d	
**30**	Yes	25 mg/wk inj	400 mg/d	3.0 g/d	LFN 10 mg^#^
**36**	Yes	25 mg/wk inj	400 mg/d	3.0 g/d	LFN 20 mg
**42**	Yes	25 mg/wk inj	400 mg/d	3.0 g/d	LFN 20 mg + gold inj 50 mg/wk
**48**	Yes	25 mg/wk inj	400 mg/d	3.0 g/d	LFN 20 mg + gold inj 50 mg/wk + Cyclosporine A 2.5 mg/kg^5#^
**54**	Yes	25 mg/wk inj	400 mg/d	3.0 g/d	LFN 20 mg + gold inj 50 mg/wk + Cyclosporine A 3 mg/kg
**60**	Yes	25 mg/wk inj	400 mg/d	3.0 g/d	LFN 20 mg + gold inj 50 mg/wk + Cyclosporine A 4 mg/kg^#^
**66**	Yes	25 mg/wk inj	400 mg/d	3.0 g/d	LFN 20 mg + gold inj 50 mg/wk + Cyclosporine A 4 mg/kg + azathioprine 1-2 mg/kg^#^
**72**	Yes	If an inadequate response has occurred after 3 months, treatment failure			

*Patients were reviewed at least every 6 weeks and therapy was increased if the treatment target had not been reached. Weeks of follow up are listed in the case of continued disease activity and hence increase in therapy at every visit. ^#^Biological DMARDs can be added, if Pharmaceutical Benefits Scheme criteria are fulfilled. ^1^If dose modification criteria (DMC) are not fulfilled, treatment is not modified; ^2^MTX administered parenterally if gastrointestinal side effects, ^3^starting dose 0.5 g/d and then increased by 0.5 g/d at weekly intervals to 2 g/d; ^4^maximum dose of MTX was based on weight and renal function: if weight <50 kg and/or creatinine clearance >30 but <60, MTX 20 mg/wk orally or parenterally, and if weight >50 kg and creatinine clearance >60 ml/min, MTX 25 mg/kg orally or parenterally. DMC, dose modification criteria; LFN, leflunomide; inj, injection.

### Dose modification criteria

Two approaches to escalating therapy were employed (criteria-based or disease activity score in 28 joints (DAS28)-based). Before 2009, dose modification was indicated if both major (swollen joint counts (SJC) >1 and elevated erythrocyte sedimentation rate (ESR) or C-reactive protein (CRP)) or one major and two minor criteria (tender joint counts (TJC) >1, morning stiffness >30 minutes, pain on visual analogue scale (VAS) >30 mm, fatigue VAS >30 mm) were fulfilled (criteria-based DMC) [2]. After 2009, the DMC were based on a DAS28 target, whereby therapy was escalated if DAS28 was >2.6 (DAS28-based DMC).

When DMC were fulfilled, dose escalation of an existing agent or addition of a new DMARD was indicated according to a structured algorithm (Table 1), unless adjustments in medication were required for toxicity. Accordingly, after maximal doses of MTX (25 mg/week via the parenteral route) and SSZ (3 g/day) were prescribed, if disease activity targets were not met, leflunomide then other DMARDs were added step by step (Table 1). After addition of leflunomide, biological DMARDs could be added if the Australian Pharmaceutical Benefits Scheme criteria were fulfilled. DMARDs were not tapered or ceased unless toxicity occurred, even if sustained remission was achieved.

### Clinic visits and assessment of disease activity

At baseline, the following information was collected: socio-demographics (age, sex, smoking status), weeks since onset of polyarthritis, shared epitope (SE), rheumatoid factor (RF) and anti-cyclic citrullinated peptide (anti-CCP) status and body mass index (BMI). At every visit, patients completed a questionnaire that assessed fatigue (assessed by 100 mm-VAS; 100 = worst fatigue), patient global assessment of disease (PGA) (100 mm VAS; 100 = worst rating), pain (100 mm VAS; 100 = worst pain), helplessness (assessed by rheumatology attitudes index (RAI)-helplessness subscale) [14] and physical function (assessed by modified health assessment questionnaire, mHAQ) [15]. A single metrologist assessed SJC and TJC, and ESR and CRP were measured. One of four rheumatologists (AL, MDWe, LGC and SMP) or a rheumatology trainee under their supervision provided an assessment of physician global assessment of disease (PhGA). Disease activity was assessed according to the DAS28 based on the ESR [16]. Quality of life (using rheumatoid arthritis quality of life questionnaires, RAQoL) [17] and radiographic status (van der Heide modified Sharp score read by blinded assessors (MDWe and SMP)) [18] were recorded annually. Data were extracted onto a structured data collection form.

### Protocol deviation

Adherence to the T2T protocol at every clinic visit over the first 3 years and the proportion of all visits associated with protocol deviation was calculated. Protocol deviations were defined according to Table 2.

**Table 2:** **Definition of protocol deviation**

**Dose escalation criteria fulfilled**	**Significant toxicity occurred** ^**1**^	**Protocol deviation**	
		**No**	**Yes**
**Yes**	No	Intensified	Continued/tapered/discontinued
**Yes**	Yes	Intensified^2^	Continued/tapered/discontinued^3^
**No**	No	Continued	Intensified/tapered/discontinued
**No**	Yes	Continued/tapered/discontinued	Intensified
**Yes/no**	Yes/no		Discontinuation of all DMARDs^4^

^1^Severe toxicities, according to physician’s assessment, deemed to be unfavourable to the health of the patient. Minor complaints were not considered as significant toxicity; ^2^when the existing drug was withdrawn or dose reduced due to toxicity but the dose of another drug was increased or a new agent was added, treatment was considered as intensified; ^3^if significant toxicity occurred; therapy escalation was not expected. However, it was considered a deviation as the disease was still active (that is, dose escalation criteria fulfilled); ^4^stopping all disease-modifying anti-rheumatic drugs (DMARDs) regardless of patient’s disease activity or toxicity status were considered deviation.

To explore whether the prevalence of protocol deviation changed over time, the prevalence in the periods between baseline and 6 months, 6 to 12 months, 12 to 24 months, and 24 to 36 months was determined. Similarly, deviations were stratified according to baseline disease activity, and patients were categorised as having LDA (DAS28 < 3.2), moderate disease-activity (MDA) (DAS28 3.2 to 5.1) or high disease-activity (had) (DAS28 > 5.1). Finally, deviations were stratified according to DAS28 remission status (that is, DAS28 < 2.6) after 3 years of treatment.

Pattern of deviations were characterized into five major categories: no deviations, temporary-occasional (deviation at just one clinic visit), temporary-relapsing (more than one deviation but not at consecutive clinic visit), persistent-occasional (deviation at more than one consecutive clinic visits occurring once) and persistent-relapsing (persistent deviation occurring more than once).

The type and nature of deviation were reported using a similar approach to that employed by Vermeer*, et al*. [9]. Accordingly, deviations were classified into one of five categories: intensifying instead of continuing; continuing instead of intensifying; tapering instead of continuing/intensifying; discontinuation instead of continuing/intensifying and any other cause. Intensifying instead of continuing means an increase in dose of an existing drug or addition of a new DMARD to the current regimen. Continuing instead of intensifying means not modifying any of the components of the existing regimen. Tapering was reducing the dose of one or more DMARDs. Discontinuation was defined as stopping one or more DMARDs for at least one clinic visit.

For each deviation, the reasons were recorded and categorised as toxicity, patient-related, physician-related, comorbidity or other causes. Toxicities were classified according to the physician’s assessment. Only significant toxicities (that is, those deemed to be unfavourable to the health of the patient) were included in this definition. These were then categorized according to the system affecte, for example, gastrointestinal, haematological, central nervous system, et cetera. Comorbidities were recorded from medical charts and physician notes. These comprised any serious and/or ongoing medical condition.

Patient-related factors were classified as poor concordance with therapy, reluctance to modify therapy and other (including social issues such as relocation or family problems). Concordance was based on patients’ self-reports [19]. It was defined as continuing the same regimen regardless of a recommendation to intensify at the previous visit, or when all or part of the medication regimen was ceased or tapered by the patient. *Reluctance to modify therapy* was defined as unwillingness to escalate the dose of existing drug or resistance to the addition of new DMARD(s) when recommended by the physician. Physician-related factors included a wait-and-see approach, limited treatment options, persistent disease and other. The wait-and-see approach applied to situations where the physician delayed therapy adjustment until the next clinic visit and waited to see if the current regimen would achieve the desired effect at this later time. Limited treatment options occurred when the options for additional therapy were limited (that is, medications in the DMARD algorithms (Table 1) were either all prescribed, contraindicated, or there was a reluctance to initiate more toxic and/or inconvenient therapies) and physicians opted to continue the current regimen. Persistent disease occurred when physicians used their clinical judgement to intensify therapy even when DMC was not fulfilled.

Not all reasons for deviation from the protocol were considered inappropriate, and reasons were considered to be permissible (generally due to toxicities and/or comorbidities) and non-permissible protocol deviations (classified as patient, physician and other causes).

### Statistical analysis

Data were analysed using Statistical Package for Social Sciences (SPSS) for Windows version 21.0 (SPSS Inc., Chicago, IL, USA). Descriptive analyses were performed for all continuous variables as mean with SD or median with IQR if the data were not normally distributed. Categorical variables were described according to the frequency and percentage. The number and percentage of visits associated with protocol deviations was determined for each patient and expressed as a total number and mean (SD) or median (IQR).

Comparison of frequency of deviation across patient groups (that is, criteria-based versus DAS28-based cohorts) and baseline disease activity (HDA versus (LDA + MDA)) was assessed using the chi-square test. Because LDA was uncommon at baseline (n = 10), this was combined with MDA for the purpose of statistical analysis. The estimate and CI for the difference in the proportion of visits with a deviation/patient for two groups was based on the Hodges-Lehman median difference. Analysis of time to first protocol deviation was based on the Kaplan-Meier estimate, and the log-rank test and Mantel-Cox *P*-values were used to identify the statistical differences between the two DMC. The Pearson product-moment correlation coefficient (for two continuous variables) and a point bi-serial correlation coefficient (for dichotomous and continuous variables) were calculated to investigate correlation between baseline factors and protocol deviations. Univariate linear regression was then performed to determine baseline factors affecting protocol deviations. Multiple linear regression analysis using the forced entry method was used to identify independent predictors of protocol deviations. All *P*-values were two-tailed and *P* <0.05 was considered statistically significant.

## Results

### Patient characteristics

Out of 231 patients who were initially considered for inclusion, a total of 198 patients completed at least one year of follow up: these were 121 patients in the criteria-based and 77 in the DAS28-based cohort. Of these, 198, 174, and 149 patients completed one, two and three years, respectively. Baseline characteristics are presented in Table 3: 33 patients were excluded due to being lost to follow up (n = 21), having short follow up (10), or other serious medical conditions (cancer and stroke, n = 2).

**Table 3:** **Baseline characteristics (n = 198 patients)**

**Female, n (%)**	**142 (71.7)**
**Age, years, median (IQR)**	56.2 (44.6 to 66.5)
**Body mass index (kg/m** ^**2**^ **), median (IQR)**	27.2 (24.0 to 30.8)
**Current and former smoker, n (%)**	107 (54.0)
**Educational status, n (%)**	
**Primary/secondary school**	79 (39.9)
**University/technical or other tertiary education**	74 (37.4)
Other*	45 (22.7)
**Engaged in paid employment, n (%)**	92 (46.5)
**Duration of polyarthritis, weeks, median (IQR)**	16 (12 to 27)
**Rheumatoid factor positive, n (%)**	124 (62.6)
**Anti-cyclic citrullinated peptide positive, n (%)**	109 (56.2)
**Shared epitope positive, n (%)**	119 (61.0)
**DAS28-erythrocyte sedimentation rate, mean (SD)**	5.5 (1.3)
**Low disease-activity, n (%)**	10 (5.1)
**Moderate disease-activity, n (%)**	63 (32.3)
**High disease-activity, n (%)**	122 (62.6)
**Physician global assessment of disease activity, median (IQR)**	54.0 (34.0 to 70.0)
**Erosive disease, erosion score ≥1, n (%)**	36 (23.5)
**Sharp/van der Heijde score, median (IQR)**	2.0 (0.0 to 7.0)
**Modified health assessment questionnaire, median (IQR)**	0.63 (0.25 to 1.13)
**Pain VAS, median (IQR)**	57.0 (30.5 to 75.0)
**Fatigue VAS, median (IQR)**	50.5 (23.0 to 69.0)
**Patient global assessment of disease activity VAS, median (IQR)**	49.0 (26.0 to 64.8)
**Rheumatology attitudes index-helplessness subscale, median (IQR)**	14.0 (10.0 to 18.3)
**Rheumatoid arthritis quality of life score, median (IQR)**	10.0 (5.7 to 15.0)
**Started triple DMARD therapy at baseline, n (%)**	175 (88.4)

*Trade school or not recorded. DAS28, disease activity score in 28 joints; DMARD, disease modifying anti-rheumatic drug; Triple DMARD therapy = methotrexate + sulfasalazine + hydroxychloroquine; VAS, visual analogue scale score.

Triple DMARD therapy containing MTX, SSZ and HCQ was the initial treatment in 175 (88.4%) patients. About 93 (47%) initiated leflunomide, 24 (12.5%) gold, 4 (2.5%) cyclosporine, 2 (1.5%) azathioprine and 14 (7.1%) biological agents, and only 5 (2.5%) were prescribed more than three DMARDs at the end of follow up.

### Prevalence of protocol deviation

**Figure 1:**
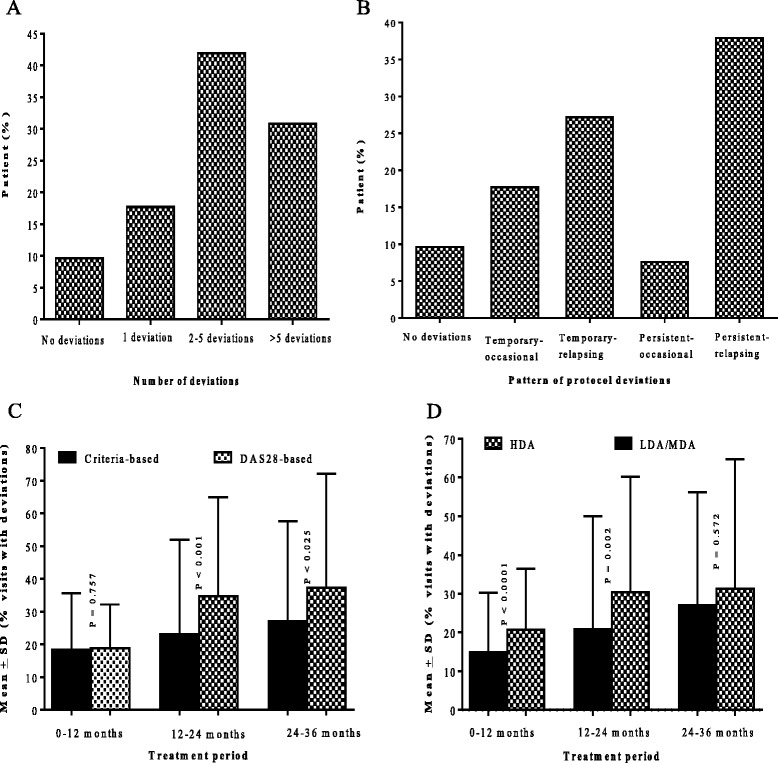
**Number and pattern of deviations and deviations according to dose modification criteria (DMC) and baseline disease activity.** Percentage of patients versus number of deviations encountered **(A)** and pattern of deviations experienced **(B)**. Mean (SD) percentage of visits with deviation per patient in each year of treatment **(C)** by criteria used to inform dose modification and **(D)** by baseline disease activity. LDA, low disease-activity; MDA, moderate disease-activity; DAS28, disease activity score in 28 joints.

There was a total of 3,654 clinic visits over the follow-up period with a mean (SD) of 18.5 (6.0) visits per patient. The mean (SD) number of clinic visits per patient during the first, second and third years was 9.8 (2.0), 5.7 (2.2) and 4.9 (1.9), respectively. Deviation from the protocol occurred in 896 of 3,654 (24.5%) visits (median of 4.0 (2.0 to 7.0) per patient). Deviation in at least one clinic visit was experienced by 179 (90.4%) of the patients (Figure 1A). Temporary deviations (n = 35) and relapsing occasional deviation (n = 54) occurred in 44.9% (89 of 198) patients. The remainder of the patients experienced either persistent (n = 15, 7.6%) or recurrently persistent deviation (n *=* 75, 37.9%) (Figure 1B).

Clinic visits were more frequent (1,225 clinic visits), but the percentage of visits with deviations was lowest (12.7%, 156 of 1,225 visits) during the first 6-months (*P* <0.0001). As treatment progressed, the percentage of visits with deviations increased to 30.6% (216 of 706 visits), 29.0% (288 of 993 visits) and 32.3% (236 of 730 visits) in the periods 6 to 12, 12 to 24 and 24 to 36 months after treatment initiation, respectively.

### Protocol deviation according to DMC

Patients treated according to the criteria-based DMC had a lower frequency of visits with a deviation compared to patients treated according to the DAS28-based protocol (23.4% versus 26.8%, median difference −4.2% (95% CI −9.02 to 0.87), *P* = 0.016). When stratifying by the period of treatment, there was no difference during the first year of therapy, but patients treated according to the DAS28-based approach experienced more deviations during the second (*P* <0.001) and third years (*P* = 0.025) (Figure 1C).

### Protocol deviation according to baseline disease activity

Patients with LDA/MDA at baseline experienced less frequent deviations compared with patients with HDA at baseline (20.1% versus 26.8%, median difference −6.3% (95% CI −11.1 to −1.3%), *P* <0.0001), and they had fewer deviations during the first (*P* <0.0001) and second (*P* = 0.002) years of treatment, but there was no difference in the third year (Figure 1D).

### Reasons for protocol deviation and time to first deviation

Patient and physician related factors were the most common reasons for protocol deviation, each accounting for 24.7% of total deviations, which were experienced by 107 (54.0%) and 103 (52.0%) patients, respectively (Tables 4 and 5). Just over half (56.7%) of protocol deviations were categorised as non-permissible.

**Table 4:** **Reasons for protocol deviation (n = 179)**

**Reason**	**Patients experiencing deviation, n (%)**	**Number of deviations**	
		**Total frequency (%)**	**Median (IQR) deviation/patient**
**Permissible**			
**Toxicities**	91 (46.0)	209 (23.3)	2 (1 to 3)
**Co-morbidities**	66 (33.3)	179 (20.0)	2 (1 to 4)
**Non-permissible**			
**Patient-related**	107 (54.0)	221 (24.7)	2 (1 to 2)
**Physician-related**	103 (52.0)	221 (24.7)	2 (1 to 3)
**Other**	35 (17.7)	66 (7.3)	1 (1 to 2)
**Total**	179 (90.4)	896 (100.0)	4 (2 to 7)

As more than one reason can contribute to a deviation, the total number of patients experiencing deviation is less than the total number of deviations.

**Table 5:** **Specific reasons for protocol deviation (n = 179)**

**Reasons**	**Patients experiencing deviation, n (%)**	**Number of deviations**	
		**Total frequency (%)**	**Median (IQR) deviation/patient**
**Toxicity**			
**Gastrointestinal**	46 (23.2)	80	1 (1 to 2)
**Haematological**	25 (12.6)	61	2 (1 to 3)
**Central nervous system**	26 (13.1)	35	1 (1 to 2)
**Abnormal liver function**	18 (9.1)	28	1 (1 to 2)
**Skin and hair**	13 (6.6)	16	1 (1 to 1)
**Fatigue**	12 (6.1)	14	1 (1 to 1)
**Muco-cutaneous**	9 (4.5)	13	1 (1 to 2)
**Other** ^**1**^	8 (4.0)	9	1 (1 to 1)
**Comorbidities**			
**Infection**	28 (14.1)	45	1 (1 to 2)
**Other co-morbidities** ^**2**^	51 (25.8)	134	2 (1 to 4)
**Patient-related**			
**Poor concordance**	71 (35.9)	106	1 (1 to 2)
**Reluctance to modify therapy**	48 (24.2)	94	1 (1 to 3)
**Other** ^**3**^	20 (10.1)	21	1 (1 to 1)
**Physician-related**			
**Wait and see**	50 (25.3)	95	1 (1 to 3)
**Limited option**	11 (5.6)	27	1 (1 to 5)
**Persistent disease** ^**4**^	14 (7.1)	15	2 (1 to 2)
**Other** ^**5**^	65 (32.8)	84	1 (1 to 2)
**Other** ^**6**^	35 (17.7)	66 (7.3)	1 (1 to 2)

^1^Respiratory, cardiovascular disease, weight loss et cetera; ^2^other muscle-skeletal conditions such as osteoarthritis and fibromyalgia, respiratory disease, cancer and pregnancy; ^3^social issues such as relocation or other problems; ^4^deviation related to persistent disease activity was intensification when dose modification criteria were not fulfilled; ^5^patient in remission, risk of toxicity, risk of flare et cetera; ^6^awaiting laboratory results, awaiting approval of biological disease modifiying anti-rheumatic drugs (DMARDs), logistic reasons, prophylaxis for tuberculosis before initiating biological DMARDs.

When patients with temporary deviations (n = 89) were compared with patients experiencing persistent or recurrent deviations (n = 90), all reasons for deviation other than physician-related factors (*P* = 0.155) were significantly associated with pattern of deviation. Accordingly, patients who experienced deviations due to toxicities (odds ratio (OR), 95% CI: 5.7 (3.0 to 10.7), *P* <0.0001), comorbidities (OR, 95% CI: 9.6 (4.8 to 21.2), *P* <0.0001) and patient-related reasons (OR, 95% CI: 3.6 (1.9 to 6.7), *P* <0.0001) were more likely to experience persistent or recurrent deviations.

**Figure 2:**
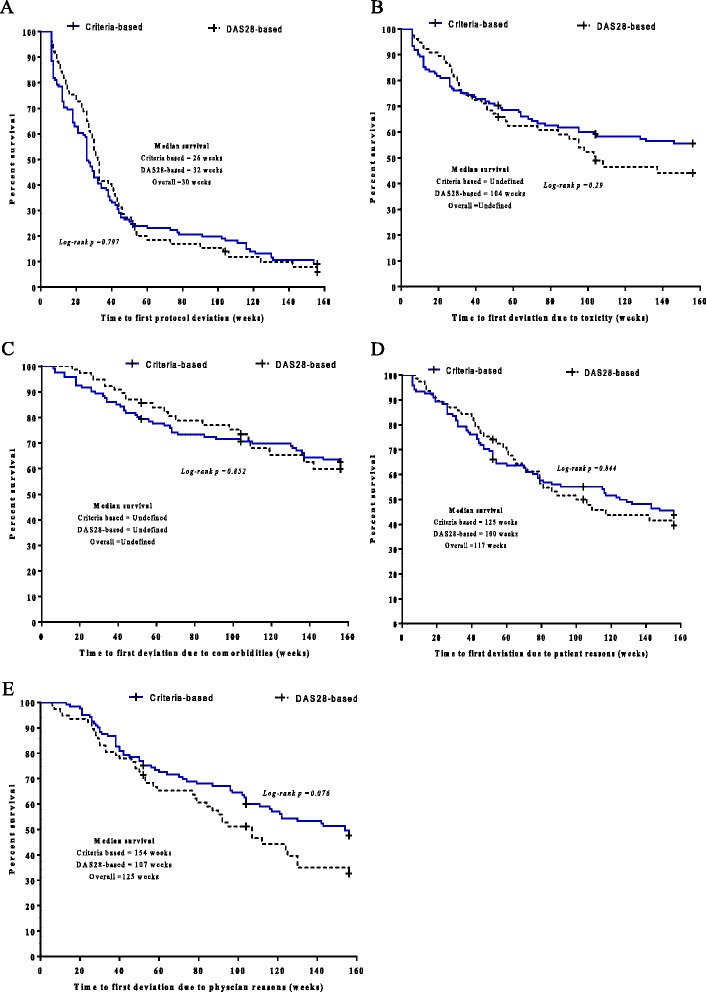
**Survival function showing time to protocol deviation.** Time to first deviations due to all reasons **(A)**, due to toxicity **(B)**, due to comorbidity **(C)**, due to patient related factors **(D)** and due to physician related factors **(E)**. Censoring of observation has occurred at either 52, 104 or 156 weeks of therapy. The overall median survival cannot be computed for toxicity and comorbidity as survival exceeds 50% at the end of the study. DAS28, disease activity score in 28 joints.

Overall, half of the patients (n = 99) experienced their first protocol deviation by week 30 [median weeks to first deviation, 95% CI = 30 (26.4 to 33.6) (Figure 2A)]. Time to first deviation was similar for patients treated according to criteria-based or DAS28-based DMC (26 *vs* 32 weeks, p = 0.797). By week 52, 147 (74.2%) had experienced at least one episode of protocol deviation. A reason-specific survival plot by DMC is presented in Figure 2B-E.

### Type of protocol deviation

Irrespective of DMC used, continuing instead of intensifying therapy was the most common type of protocol deviation (59.9%, 537 of 896 deviations, Table 6).

**Table 6:** **Type of protocol deviation**

**Type**	**Number of deviations**		**Total**	***P*** **-value** ^**#**^
	**Criteria-based**	**DAS28-based**		
**Continued rather than escalating**				
**Toxicity**	33	26	59	
**Patient-related**	92	63	155	
**Physician-related**	83	60	143	
**Comorbidity**	79	42	121	
Other*	42	17	59	
**Total, n (%)**	329 (58.1)	208 (63.0)	537 (59.9)	0.005
**Tapered rather than continuing/escalating**				
**Toxicity**	31	22	53	
**Patient-related**	25	13	38	
**Physician-related**	11	12	23	
**Comorbidity**	4	3	7	
Other*	1	0	2	
**Total, n (%)**	72 (12.7)	50(15.2)	122 (13.6)	0.077
**Discontinued rather than continuing/escalating**				
**Toxicity**	68	28	96	
**Patient-related**	20	5	25	
**Physician-related**	0	0	0	
**Comorbidity**	37	14	51	
**Other**	0	0	0	0.076
**Total, n (%)**	125 (22.1)	47 (14.2)	172 (19.2)	
**Dose escalated rather than continuing**				
**Toxicity**	0	0	0	
**Patient-related**	2	0	2	
**Physician-related**	29	20	49	
**Comorbidity**	0	0	0	
Other*	2	1	3	
**Total, n (%)**	33 (5.8)	21 (6.4)	54 (6.1)	0.402
Other**				
**Toxicity**	1	0	1	
**Patient-related**	0	1	1	
**Physician-related**	3	3	6	
**Comorbidity**	0	0	0	
Other*	3	0	3	
**Total, n (%)**	7 (1.3)	4 (1.2)	11 (1.2)	0.843
**Total deviation**	566	330	896	0.016

*Examples of other type of protocol violation include awaiting laboratory results, awaiting approval of biological disease modifying anti-rheumatic drugs, prophylaxis for latent tuberculosis before initiating biologics, and other logistic reasons or unknown reason. ^#^Statistical tests based on the chi-square test compared the frequency of responses (clinic visits with deviation versus no deviation) between patient groups due to specific types of deviation. **Unknown, addition of very high unusual dose or intensification of two or more drugs at a time, intensifying instead of tapering/discontinuing.

Patients with HDA at baseline had a higher rate of deviation due to continuing instead of escalating therapy (*P* <0.0001) and discontinuing instead of continuing/escalating (*P* =0.014) compared to those with LDA/MDA at baseline.

### Protocol deviation according to treatment response

**Figure 3:**
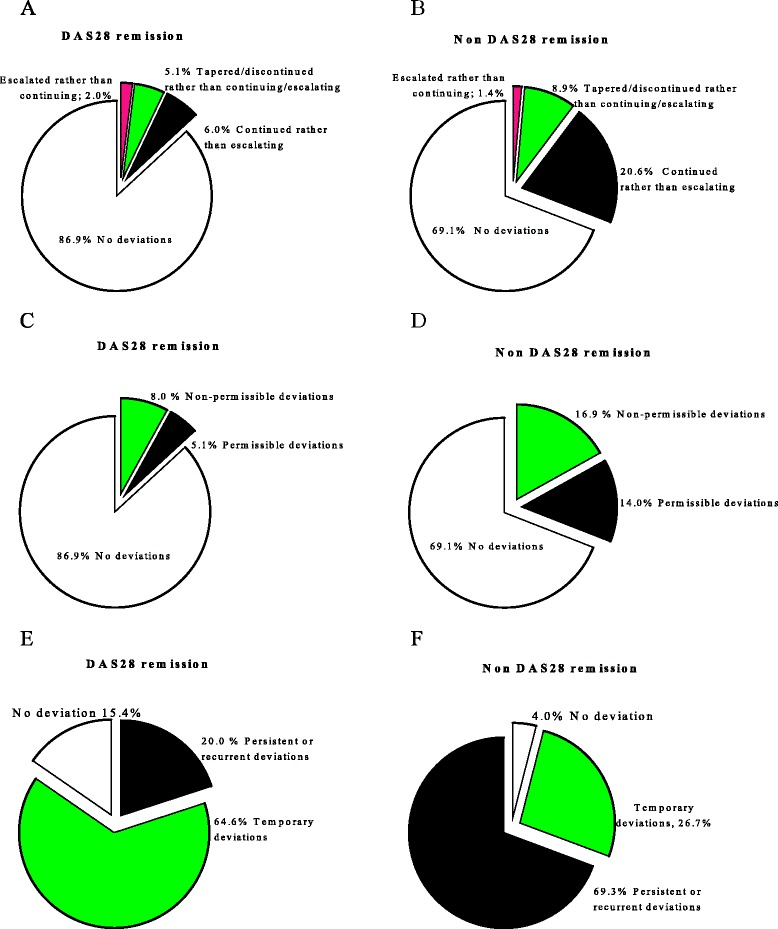
**Protocol deviations according to treatment outcomes.** Deviations by type **(A, B)**, reason for deviations **(C, D)** and proportion of patients facing different patterns of deviations according to disease activity in 28 joints (DAS28) remission status **(E, F)**.

Remission according to DAS28 (DAS28 < 2.6) was achieved by 28.5% (43/152), 42.3% (77/182), 46.3% (76/164) and 46.4% (65/140) at 6 months, 12 months, 24 months and 36 months, respectively. Remission rates were higher in patients with baseline LDA/MDA, as compared with HDA, at 6 months (47.2 versus 17.3%, *P* <0.0001) and 12 months (54.0 versus 36.1%, *P* = 0.021). At 24 and 36 months, however, differences did not reach statistical significance (54.4 versus 42.1%, *P* = 0.132 at 24 months and 55.3 versus 42.4%, *P* = 0.148 at 36 months). There were more clinic visits for patients not in DAS28 remission compared with those in DAS28 remission (mean (SD): 22.6 (4.7) versus 18.9 (3.6)). The prevalence of protocol deviation was lower among patients who achieved remission (13.1%; 162 deviations out of 1,228 visits) compared with those who did not (30.9%; 523/1692) (*P* <0.0001) (Figure 3A and B). In both groups, non-permissible deviations were more common than permissible deviations (that is, toxicity and comorbidity); however, the proportion of deviations that were non-permissible was lower among patients who achieved remission compared with those who did not (8.0 versus 16.9%, *P* <0.0001) (Figure 3C and D).

The proportion of patients with a persistent/recurrent deviation was significantly less in patients who achieved remission as compared to patients who did not (20.0% versus 69.3%, *P* <0.0001) (Figure 3E and F). Patients in remission had a lower prevalence of deviations due to toxicity (12.1 versus 34.3%, *P* <0.0001), comorbidities (12.1 versus 27.1%, *P* = 0.003), patient-factors (15.7 versus 37.9%, *P* <0.0001) and other reasons (4.3 versus 15.7%, *P* = 0.003), but deviations secondary to physician related factors were not different between patients who did, and did not achieve remission (23.6 versus 33.6%, *P* = 0.156).

### Baseline predictors of protocol deviation

The patient-reported outcome measures including mHAQ *(r* = 0.22, *P* = 0.002), pain *(r* = 0.18, *P* = 0.015), fatigue *(r* = 0.21, *P* = 0.004), helplessness *(r* = 0.28, *P* <0.0001) and PGA *(r* = 0.24, *P =* 0.001), and paid employment *(r* = −0.182, *P =* 0.010) were correlated with the total number of protocol deviations. Body mass index (*r* = 0.29, *P* <0.0001), baseline DAS28 *(r* = 0.18, *P* = 0.028), and TJC *(r* = 0.21, *P* = 0.003) were also associated with the number of deviations. BMI, DAS28, TJC, mHAQ, PGA, fatigue and helplessness were associated with permissible deviations, whereas BMI and helplessness were the only baseline variables that were associated with non-permissible deviations. Using multivariate analysis, BMI (β, 95% CI 0.78 (0.28 to 1.27), *P* = 0.003) and helplessness (β, 95% CI 0.77 (0.03 to 1.50), *P* = 0.04) remained independent predictors of protocol deviations, but the predictive power of the model was not strong (*R*^2^ = 0.17).

## Discussion

In the context of routine clinical practice, deviations from a T2T strategy according to predefined DMC for a cohort of patients with recent onset RA occurred in approximately 25% of clinical visits. Of these deviations, 43.3% were justifiable on clinical grounds. The most common type of protocol deviation was failure to escalate dose when indicated, which accounted for more than half of deviations.

The need for early referral and commencement of DMARDs followed by close monitoring of disease activity requires frequent patient visits, which may be a barrier to the T2T approach in clinical practice [20]. In our cohort, patients attended an average of 6.2 and 9.8 visits in the first 6 and 12 months following DMARD initiation respectively, and as such, the need for frequent visits did not appear to be an issue. However, 21 individuals were lost to follow up. The concordance with T2T may have been different in these patients for the same reasons they failed to continue attending the clinic. As the aim of this article was to evaluate ongoing issues with implementing T2T strategy, we excluded these patients from current analysis.

Frequent dose intensification shortly after DMARD initiation was particularly common in the DAS28-based DMC group, which may have been due to increased physician awareness of the importance of early intensive management in recent years and/or the greater conviction imbued by a well-recognised composite measure of disease activity. Furthermore, compared with the criteria-based DMC cohort, approximately 20% more visits fulfilled the criteria for increasing therapy when the DAS28-based DMC was used (data not shown). These latter patients, therefore, progressed through the algorithm more quickly, reaching the less attractive drug options (that is, gold, cyclosporine and azathioprine) earlier, which may be associated with more deviations (see Figure 1) due to a reluctance to initiate medications that were perceived to be more toxic, particularly after the first year. Also, with more prolonged treatment there may be a greater likelihood that the patient and possibly the physician will be more tolerant of the status quo and less demanding in their quest for solutions. Protocol deviation was also higher among patients with HDA at baseline. These patients experienced more continuation rather than escalation of therapy, which may have been due to their experiencing more side effects from medications (data not shown). The higher rate of deviation in patients with HDA at baseline was contrary to our expectation that there would be a high incidence of (rapid) intensification instead of continuation when compared to patients with LDA/MDA. This impact of baseline disease activity, however, declined over time. This was in line with the influence of baseline disease activity on treatment outcome, which also declined as treatment progressed. The higher prevalence of deviation and the lower rate of remission in patients with baseline HDA indicate the extent to which protocol deviation translates into treatment outcome.

In the DREAM remission-induction cohort, deviation in at least one clinic visit was similar to our cohort (91.0 versus 90.4%), but the overall prevalence of deviations in our cohort was lower (24.5% versus 30.7%) [9]. Furthermore, the rate of deviations in those who achieved remission was 13.1% in this cohort compared to 19.0% in the DREAM cohort, while in those who did not achieve remission the corresponding rates were 30.9% and 42.1% [9]. Difference in clinical settings, the nature of protocol used (for example, biological DMARDs were used more commonly in the DREAM cohort), patient characteristics or cultural reasons may have resulted in differences in the prevalence rate. Furthermore, the fact that there were stable personnel in our cohort (for example, physicians and a metrologist with standardisation of application of the T2T protocol), may also explain the lower rate of deviation in this study.

This study differs from earlier reports in three major respects. First, long-term (up to three years) follow up in compliance with the T2T approach is reported, which allowed identification of the stages of therapy when deviation is more likely to occur, and the impact of duration of therapy on the type of deviation that is likely to occur.

Second, we studied the pattern of protocol deviations, which appears to have an important relationship with outcome. For example, delaying a suggested recommended change to therapy to the next visit (that is, temporary deviations) may have little impact on the overall treatment outcome. Furthermore, the prevalence of persistent/recurrent deviations was low among patients in remission compared with those in non-remission whose disease activity dictated more frequent adjustments in treatment (Figure 3E). Deviations due to comorbidity, toxicity or patient-related factors were more likely to have persistent deviations, whilst physician-related factors did not differ between temporary and persistent deviations. Interestingly, these relations also extended to treatment outcome; DAS28 remission was less likely among patients experiencing deviations due to toxicity, comorbidity, patient-related factors or other factors, whereas physician-related factors did not have any impact on long-term treatment outcome.

Finally, we also explored whether baseline characteristics would predict the occurrence of protocol deviations, but the only objective baseline factor associated with protocol deviation was BMI. This has potential long-term clinical implications. Increased BMI is correlated with physical inactivity and greater risk of comorbid conditions including diabetes, hypertension, cardiovascular disease and other musculoskeletal symptoms, which may also affect how RA is treated [21,22]. Other objective measures such as baseline radiographic scores, laboratory values as well as socio-demographic variables, including gender, age and smoking habits, did not predict protocol deviation. Most of the factors correlating with protocol deviations in a univariate analysis, albeit weakly, were patient-reported outcomes, which may not directly measure the pathophysiological status of disease and can be biased by other comorbid conditions [23]. On the other hand, decisions about dose modification could be influenced by patient-reported outcomes to the extent that they provide an indication of the life impact of disease and personal factors such as illness behaviours. Furthermore, the effect of patient reported outcomes such as helplessness could be mediated through several factors. For example, patients with high levels of helplessness are known to experience higher levels of anxiety, depression, low self-esteem, impaired activities of daily living, low socioeconomic status and more symptoms such as fatigue, pain and stiffness [24-26]. This may in turn lead to poor concordance with therapy and/or reduce patient motivation to comply with a physician’s recommendation.

### Toxicities and comorbidities

Toxicity can restrict long-term utilization of DMARDs [27] and accounted for 16.6% of total deviations in this cohort (Table 6). After DMARD-induced toxicity, judicious re-challenge or addition of new drugs should be a gradual and individualised process to avoid recurrence of unwanted effects. Gastrointestinal side effects, such as nausea, vomiting, abdominal pain/cramps, anorexia and diarrhea, were the leading drug-related safety issues responsible for deviations. These side effects are common with all conventional DMARDs [28], in particular sulfasalazine, which has been previously reported in this cohort [29]. In addition, comorbidities are very common in patients with RA [30], and can impact on physical function [31]. These comorbidities may be independent of or related to RA and its treatment, and it is often difficult to discriminate between these causes [32], but regardless of this, withholding or reducing DMARDs until these conditions have resolved was considered to be a justifiable reason for protocol deviation.

### Patient and physician point of view

In the current study, both patients and physicians contributed equally to failure of adherence to a strict T2T strategy. According to international T2T taskforce recommendations [6], the management of RA should be based on a ‘shared decision between patient and physician’, and effective implementation is based on patients’ long-term commitment to therapy. However, full commitment was not demonstrated in this study, as about one quarter of all deviations (24.7% of total visits associated with deviations) were associated with patient-related reasons, including unwillingness to escalate their current treatment and poor concordance with previously agreed therapy. While concordance was not evaluated systematically in this study, patient self-reported concordance with therapy was used as a rough estimate of concordance rate, although this is likely to overestimate the true concordance level [19]. Poor concordance occurred when patients remained on the same dose regardless of a recommendation to intensify at the previous visit, or when all or part of the medication regimen was ceased or tapered by the patient. If this occurred, the physician had no choice but to reinstate the earlier regimen instead of intensifying, even when dose escalation was indicated. Systematically assessing the extent of concordance with therapy in the context of T2T and implementing tailored approaches to improving concordance with therapy is almost certainly critical to the success of this strategy.

Reluctance to modify therapy when indicated is a common phenomenon in RA. Wolfe and Michaud [11] reported that a common preference of patients undergoing long-term treatment for RA was maintenance of their current status and treatment, rather than striving for longer-term benefits with more intensive treatment, with attendant risks of new toxicities [11]. Van Hulst *et al.* [33] reported that factors that patients use to inform this decision are different from those used by clinicians. Current disability status, motivation to get better, belief in their physician, satisfaction with their current medications, and current number of painful joints affect patients’ decision to modify therapy [33]. Patients may make their own risk-benefit assessment before making decisions about their therapy, and therefore may need to be educated about the importance of short-term disease control in improving long-term treatment outcomes.

It may be difficult in practice to strictly comply with rigid T2T principles given the heterogeneity of patient populations and a tendency to individualise the approach. For example, sometimes therapy was intensified if there was a belief that disease was still active, even if a DMC was not fulfilled. Similarly, components of DAS28 such as ESR are not specific for RA disease activity, and disease may be considered inactive if elevated ESR was thought to be due to an unrelated cause. Such decisions based upon clinical judgement rather than composite disease activity measures are common practice [9,34,35], and may be considered rational and acceptable by physicians, but were considered unacceptable protocol deviations in this study. Another common approach practised by physicians is the so-called wait-and-see policy, where the physician thinks more time is needed for the drug to deliver optimal benefit and dose escalation is delayed until the next visit and beyond so as to minimise potential overtreatment. This was considered an unacceptable deviation in this study, and was especially common with leflunomide and gold injections, although the latter can take up to 6 months to produce maximal benefit [36].

The results of the current study should be interpreted within the following methodological limitations. The data for this analysis were compiled from various sources including physicians’ correspondence, a database containing information on patient characteristics, treatments and outcomes and from other medical records, but complete information from all visits may not be available. The frequency of clinic visits varied between patients depending on disease activity, and if patients failed to attend scheduled visits. In the case of the latter, non-concordance with the T2T protocol could have been underestimated. Our assessment of reasons for deviations was subjective and sometimes reasons for deviations were overlapping/inter-related, and as a result there is a potential for misclassification bias, including categorisation as permissible or non-permissible deviations. While we explored comorbidities as a reason for deviation, patient baseline comorbidities and their impact were not systematically assessed. For example, we did not evaluate the extent to which the treatment protocol was difficult to follow among patients with multiple comorbidities. Various clinical guidelines that utilise T2T differ from each other in certain aspects [10]. Because this study was conducted in the context of local T2T guidelines, which are based on parallel triple DMARD therapy, the findings may not be generalizable to other treatment approaches such as step-down, step-up or monotherapy. Despite these limitations, the study addressed important issues concerning the extent of deviation from T2T recommendations, reasons for deviations, whether deviations are justifiable or not and baseline factors affecting deviation. Further work towards understanding how T2T operates in daily practice and the relationship between compliance with treatment guidelines and short- and long-term treatment outcomes is required. Other unanswered questions in the setting of DMC being fulfilled include whether either a new medication or increasing the dose of an existing drug is more likely to be associated with protocol deviation, the type of medication most likely to contribute to deviations and whether strict adherence to treatment guidelines, particularly early in the course of disease, will prevent patients from requiring expensive biological agents.

## Conclusion

In conclusion, there was a low level of protocol deviation when a T2T strategy was implemented in real-life clinical practice. Just under half of the observed deviations were considered permissible and were related to either drug toxicity or patient comorbidities. Patients with high baseline BMI and helplessness scores were more likely to experience protocol deviations.

## Abbreviations

ACR: American college of rheumatology
anti-CCP: anti-cyclic citrullinated peptide
BMI: body mass index
CRP: C-reactive protein
DAS28: disease activity score in 28 joints
DMARDs: disease-modifying anti-rheumatic drugs
DMC: dose modification criteria
ESR: erythrocyte sedimentation rate
EULAR: European league against rheumatism
HCQ: hydroxychloroquine
HDA: high disease-activity
LDA: low disease-activity
MDA: moderate disease-activity
mHAQ: modified health assessment questionnaire
MTX: methotrexate
OR: odds ratio
PGA: patient global assessment of disease
PhGA: physician global assessment of disease
RA: rheumatoid arthritis
RAI: rheumatology attitudes index
RAQoL: rheumatoid arthritis quality of life
RF: rheumatoid factor
SE: shared epitope
SJC: swollen joint counts
SSZ: sulfasalazine
T2T: treat-to-target
TJC: tender joint counts
VAS: visual analogue scale
